# A Polymerization-Associated Structural Switch in FtsZ That Enables Treadmilling of Model Filaments

**DOI:** 10.1128/mBio.00254-17

**Published:** 2017-05-02

**Authors:** James M. Wagstaff, Matthew Tsim, María A. Oliva, Alba García-Sanchez, Danguole Kureisaite-Ciziene, José Manuel Andreu, Jan Löwe

**Affiliations:** a MRC Laboratory of Molecular Biology, Cambridge, United Kingdom; b Centro de Investigaciones Biológicas, CSIC, Madrid, Spain; Duke University School of Medicine

## Abstract

Bacterial cell division in many organisms involves a constricting cytokinetic ring that is orchestrated by the tubulin-like protein FtsZ. FtsZ forms dynamic filaments close to the membrane at the site of division that have recently been shown to treadmill around the division ring, guiding septal wall synthesis. Here, using X-ray crystallography of *Staphylococcus aureus* FtsZ (SaFtsZ), we reveal how an FtsZ can adopt two functionally distinct conformations, open and closed. The open form is found in SaFtsZ filaments formed in crystals and also in soluble filaments of *Escherichia coli* FtsZ as deduced by electron cryomicroscopy. The closed form is found within several crystal forms of two nonpolymerizing SaFtsZ mutants and corresponds to many previous FtsZ structures from other organisms. We argue that FtsZ’s conformational switch is polymerization-associated, driven by the formation of the longitudinal intersubunit interfaces along the filament. We show that such a switch provides explanations for both how treadmilling may occur within a single-stranded filament and why filament assembly is cooperative.

## IMPORTANCE

The FtsZ protein is a key molecule during bacterial cell division. FtsZ forms filaments that organize cell membrane constriction, as well as remodeling of the cell wall, to divide cells. FtsZ functions through nucleotide-driven filament dynamics that are poorly understood at the molecular level. In particular, mechanisms for cooperative assembly (nonlinear dependency on concentration) and treadmilling (preferential growth at one filament end and loss at the other) have remained elusive. Here, we show that most likely all FtsZ proteins have two distinct conformations, a “closed” form in monomeric FtsZ and an “open” form in filaments. The conformational switch that occurs upon polymerization explains cooperativity and, in concert with polymerization-dependent nucleotide hydrolysis, efficient treadmilling of FtsZ polymers.

## INTRODUCTION

FtsZ is an ancient, filament-forming, tubulin-like GTPase protein found in the vast majority of bacteria and archaea, where it acts as a central component of the cell division machinery (1–3). FtsZ is localized to the plasma membrane at future division sites, resulting in the emergence of a ring structure around the center of the cell, the Z ring. FtsZ is anchored to the plasma membrane by other proteins, most often FtsA but also ZipA and/or SepF (4–6). FtsA is a divergent actin homologue that forms copolymers with FtsZ and contains an amphipathic helix that facilitates membrane attachment (7).

After the localization of FtsZ, a large number of other proteins are recruited to the division site. These proteins carry out remodeling and synthesis of cell wall during the division process. Together, these proteins have been termed the divisome, although it is not known whether there is a stable multisubunit complex at the heart of the divisome.

The precise molecular architecture of the Z ring remains unclear, although it is probably composed of dynamic overlapping filaments along the circumference of the ring, at least during the later stages of the division process in rod-shaped model organisms such as *Escherichia coli* (8). It was already clear from early fluorescence microscopy studies that during the cell division process, the Z ring contracts with the constricting septum (9). *In vitro* reconstitution experiments of FtsZ and FtsA with membranes showed that these two components alone deform membranes (8, 10). Together with homology to force-generating eukaryotic tubulins, this prompted the suggestion that FtsZ has a role in generating forces required for constriction. In contrast, observations of constrictions and divisions of cells with helical Z rings, incomplete Z rings, and divisomes with modified FtsZ properties, support the opposing idea that FtsZ does not provide an indispensable driving force for constriction (11–14). The alternative candidate for force generation is cell wall remodeling. A third option is that cell wall remodeling and Z-ring dynamics are interlinked processes that work together from the inside and outside to generate the forces needed for division to occur robustly and efficiently under many circumstances.

Treadmilling is a property of certain cytomotive filaments where the strength of intersubunit interfaces is made dynamic in time through nucleotide hydrolysis, which is, in turn, triggered by the polymerization reaction. Treadmilling, characterized by preferential growth at one filament end and loss at the other, requires a difference in the rate of net polymerization and depolymerization at the so-called plus and minus ends of the filaments (such that filaments have a kinetic polarity, as well as a structural polarity).

Recently, *in vitro* treadmilling of FtsZ filaments has been reported on supported bilayers with (15) and without FtsA (16) and also *in vivo* where FtsZ filaments were found to treadmill with components of the divisome around the division site (17, 18). These findings have resurrected an old model of bacterial cell division: the template model, in which the closing septum constricts by new cell wall material being deposited in concentric rings on the inside of old material by moving synthesis machinery, which in turn is guided or organized into a ring by dynamic FtsZ filaments. This idea fits into the third category of ideas listed above about the role of FtsZ, i.e., FtsZ dynamics and cell wall synthesis working together to facilitate constriction.

FtsZ so far was not considered a good candidate for treadmilling behavior, largely because it is not known if any functionally relevant structures are formed in cells beyond single-stranded protofilaments (8, 19). It is difficult to explain how the kinetic polarity needed for treadmilling can be coupled to the structural polarity of single-stranded filaments, such as those of FtsZ (20). Given that treadmilling is seen in multiple organisms *in vivo*, and *in vitro*, including observation of single protofilaments treadmilling *in vitro* (15), we conclude that treadmilling is an intrinsic property of FtsZ filaments.

Surprisingly, knowledge of FtsZ filament structure is limited. Only one FtsZ crystal form, from *Staphylococcus aureus* FtsZ (SaFtsZ; PDB IDs 3VOA and 3VO8), has revealed a straight protofilament of FtsZ, as might be expected from electron micrographs of many different FtsZ filaments and by analogy to eukaryotic tubulins (21). The conformation of SaFtsZ subunits in those straight filaments showed an unusually (compared to FtsZ structures from other organisms) open conformation, with the N-terminal GTP binding domain (NTD) and the C-terminal GTPase activation domain (CTD) being rotated and shifted apart (~27° compared to, e.g., *Bacillus subtilis* structure PDB ID 2VAM). Subsequent crystallization efforts using SaFtsZ constructs with large changes to the critical T7 loop that normally contacts the GTP/GDP nucleotide bound to the next subunit were successful in generating crystals where SaFtsZ adopted a different “closed” conformation more similar to that of FtsZ proteins from other species (PDB IDs 3WGK and 3WGL) (22). These crystals also contained straight protofilaments. It remains unclear what causes the conformational switch seen in these T7 mutant proteins. It is possible that the switch was promoted by nonspecific crystal contacts or by the alterations of the T7 loop. Also unknown is whether unmodified SaFtsZ can adopt a closed conformation.

SaFtsZ has also been crystallized in an open conformation in complex with the FtsZ functional inhibitor and filament stabilizer PC190273 (PDB IDs 3VOB and 4DXD). The drug is bound in the cleft between the NTD and the CTD, which is only possible in the open conformation, suggesting that the mechanism of drug action is to lock the protein in this state (21, 23, 24). Isolated, open-form SaFtsZ monomers relax into the closed conformation during molecular dynamic simulations (25). Fluorescent analogues of PC190723 have recently been used to monitor apparent opening and closing of the interdomain cleft in solution as a function of the FtsZ polymerization state (26). Together, these results hint that the closed form of different FtsZ proteins seen in many crystals is the predominant conformation of monomeric FtsZ proteins and vice versa that filamentous FtsZ in solution is in the open conformation seen in SaFtsZ filament crystals. What is lacking is robust structural evidence that this is the case.

FtsZ has two properties in common with actin and tubulin that until now have been hard to explain. First, FtsZ exhibits cooperative assembly, with a critical concentration and a lag phase in assembly. This is not possible for a single-stranded, isodesmic filament with rigid subunits, and an assembly switch has long been hypothesized to explain this cooperativity (27). Second, filament treadmilling is presumed to require multistrandedness (28), which FtsZ may not display *in vivo*.

Here we demonstrate that FtsZ proteins do indeed undergo a conformational switch, that this switch is associated with polymerization and not the nucleotide hydrolysis state, and that switching provides a possible mechanism both for cooperative assembly and for single-protofilament treadmilling, which has been proposed to be the key dynamic filament behavior used to organize cell wall remodeling.

## RESULTS AND DISCUSSION

### SaFtsZ T66W and F138A are polymerization and GTPase compromised.

Currently, there is no pair of structures showing an unmodified FtsZ molecule in the closed and open states, as discussed above. We set out to generate an SaFtsZ structure with the molecule in the closed form, which we suspected to be found in monomeric FtsZ proteins, by introducing single point mutations inhibiting polymerization in regions of the structure thought to be far away from regions involved in nucleotide binding or conformation switching. Specifically, two SaFtsZ mutations, F138A and T66W, were designed to inhibit SaFtsZ filament formation on the basis of equivalent mutations inhibiting the assembly of *Methanocaldococcus jannaschii* FtsZ (M164A [29] and T92W [30], respectively; polymerization inhibition of T92W [unpublished data]). Both mutation sites are located on the “top” surface of FtsZ, in the N-terminal GTP-binding domain, and are part of the longitudinal protofilament interface seen in crystals.

**FIG 1:**
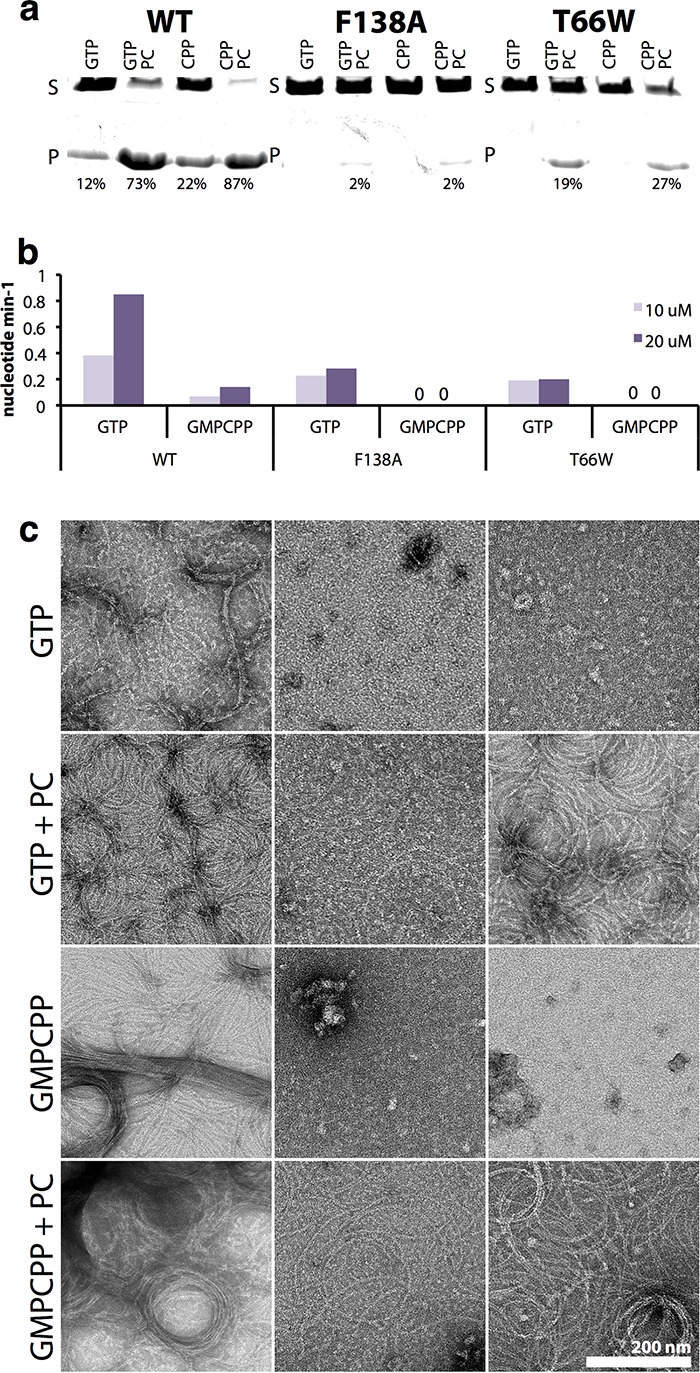
FL T66W and F138A mutant SaFtsZ proteins have compromised polymerization and GTPase activities. (A) Polymerization of FtsZ proteins at 10 μM was assayed by sedimentation in the presence of GTP and GMPCPP (CPP) with and without the FtsZ inhibitor PC190723 (PC). Pelleted (P) and soluble (S) protein samples were subjected to SDS-PAGE in the same gel lane with a delay. The percentage of pelleted protein was estimated from integration of band intensities. (B) GTPase activity of FtsZ proteins at 10 and 20 μM in the presence of GTP or GMPCPP. (C) Polymerization of FtsZ proteins in the presence of GTP and GMPCPP with and without the FtsZ inhibitor PC190723 (PC) was assessed by negative-stain electron microscopy. All images are at the same magnification (scale bar, 200 nm). WT, wild type.

Full-length (FL), untagged wild-type and F138A and T66W mutant SaFtsZ proteins were purified and characterized biochemically (Fig. 1). Filament formation in both mutated SaFtsZ proteins was compromised, since no filament formation was detected by sedimentation (Fig. 1A) or negative-stain electron microscopy (Fig. 1C) for either T66W or F138A in the presence of GTP or guanosine-5′-[(α,β)-methyleno]triphosphate (GMPCPP), a slowly hydrolyzable analogue of GTP. FtsZ GTPase activity is largely dependent on polymerization as one subunit provides catalytic residues to the active site of the next subunit through residues in loop T7. Both mutants have weak GTPase activity (Fig. 1B), indicating that monomers may at least associate to form transient but functional active sites. In support of this, on addition of PC190723, the mutant proteins did form filaments detectable by sedimentation and electron microscopy in the presence of GTP and GMPCPP. We conclude that SaFtsZ T66W and F138A are polymerization and GTPase compromised but retain some residual activities.

### SaFtsZ adopts either a closed or an open conformation.

**FIG 2:**
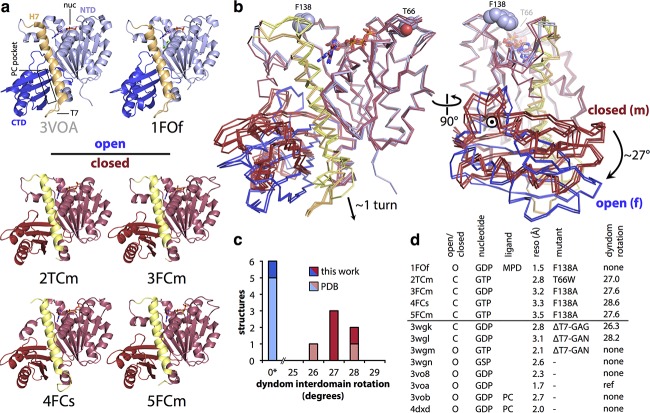
Nucleotide-bound SaFtsZ crystal structures group into two conformations, open and closed. (A) The five SaFtsZ (TR to residues 12 to 316) structures determined here and PDB ID 3VOA are shown in cartoon representations with nucleotides as sticks colored by element. The 2-methyl-2,4-pentanediol (MPD) molecule in 1FOf is shown as green sticks. The structures are colored according to conformation. Closed structures are red, with the N-terminal GTP-binding domain in light red and the C-terminal GTPase activation domain in dark red, and the central helix, H-7, is highlighted is yellow. Open structures are blue, with the NTD in light blue and the CTD in dark blue, and the central helix H-7 is highlighted in orange. All structures shown in the same orientation, after alignment with the NTD of 3VOA (residues 13 to 165). The 4FCs domain-swapped pseudomonomer is formed of two polypeptides. Note the different positions of the CTD in the two sets of structures. The position of the PC190723 binding pocket is indicated on the 3VOA molecule. (B) Superposition of the six structures in panel A shown in Cα ribbon representation after alignment as for panel A, with the same color scheme. Nucleotides are shown as sticks. Side chains of residues F138 and T66 of the wild-type structure are shown as spheres, and noncarbon atoms are colored by element (left). The same view as in panel A (right) with molecules rotated 90° as indicated. The axis of interdomain rotation is indicated by the circled dot and the curved arrows. (C, D) Census of available nucleotide-bound SaFtsZ structures. (C) Bar graph indicating that DynDom, model-free assessment of dynamic protein domains, essentially produces two groups when SaFtsZ structures are compared to PDB ID 3VOA (no interdomain rotation or an ~27° shift around the axis in panel B, right). (D) Table with information about nucleotide-bound SaFtsZ structures. The horizontal line separates the structures determined here (top) from previous structures in the PDB. PC, PC190723; reso, resolution.

We solved five crystal structures of the globular domains of SaFtsZ T66W and F138A (Fig. 2; see Table S1 in the supplemental material). SaFtsZ constructs truncated (TR) to residues 12 to 316 were used to remove the N- and C-terminal tails of FtsZ previously found to inhibit crystallization. For easier reference, the five SaFtsZ structures are named herein in the form #XXx: number (1 to 5), mutation (F for F138A, T for T66W), monomer conformation (O for open, C for closed), and finally the arrangement of monomers within the crystal (m for monomeric, f for filamentous, single protofilament and s for split/domain swapped).

10.1128/mBio.00254-17.2 TABLE S1  Crystallographic and cryoEM data obtained in this study. Download TABLE S1, DOCX file, 0.1 MB. Copyright © 2017 Wagstaff et al. 2017 Wagstaff et al. This content is distributed under the terms of the Creative Commons Attribution 4.0 International license.

One structure, 1FOf, was in the open form and essentially identical (crystallographically isomorphous) to previously published wild-type SaFtsZ open conformation structures (Cα root mean square deviation [RMSD] versus PDB ID 3VOA, 0.33 Å) (Fig. 2A, top). FtsZ molecules in 1FOf form completely straight single-stranded filaments (protofilaments) with a 44-Å repeat extending throughout the crystal. Four of the polymerization compromised FtsZ point mutant structures (2TCm, 3FCm, 4FCs, and 5FCm) were in the closed conformation previously seen in SaFtsZ after extensive mutation of the T7 loop (e.g., Cα RMSD 2TCm versus PDB ID 3WGL, 1.50 Å). Indeed, the closed structures were successfully solved by molecular replacement with one of the previous T7 loop replacement mutant structures (PDB 3WGL) as the starting search model. Unlike the closed-form T7 mutant SaFtsZ crystals, none of our closed crystal forms contain straight filaments running through the crystals.

When we analyzed the conformations of all of the available nucleotide-bound SaFtsZ structures, it became clear that they do fall into two discrete groups (Fig. 2B to D). We excluded SaFtsZ apo structures (e.g., PDB ID 3VO9), which are very different and are unlikely to be physiologically relevant given the high concentration of guanine nucleotides in cells. The two conformations, open and closed, can be distinguished clearly by the change in the interdomain angle between the NTD and the CTD. If we consider the NTD to be fixed in space, the switch to the open conformation is best defined (as determined by the model-free algorithm implemented in the program DynDom [31]) as an ~27° rotation of the CTD versus the closed conformation around an axis of rotation, as indicated by the circled dot in Fig. 2B, right. This rotation is accompanied by a downward shift of the central helix 7 (H-7, yellow in Fig. 2) by almost one helical turn (Fig. 2B, left).

It is important to note that, including previous work, we now have available SaFtsZ structures with all permutations of open and closed conformations and bound GTP/GDP nucleotide, making it difficult to imagine that the open or closed conformational state and the nucleotide state are linked. Also, for the first time, with this work, we have structures showing a single nearly native FtsZ molecule in multiple conformations: SaFtsZ F138A crystallized in the open conformation in straight filaments as 1FOf, in the closed form as a monomer in two different space groups in 3FCm and 5FCm, and as a split/domain-swapped closed-form monomer in 4FCs. Domain swapping has been seen before in FtsZ (32) and highlights the surprising independence of the NTD and the CTD. This makes it unlikely that the conformational change seen is caused by modifications made to the protein.

**FIG 3:**
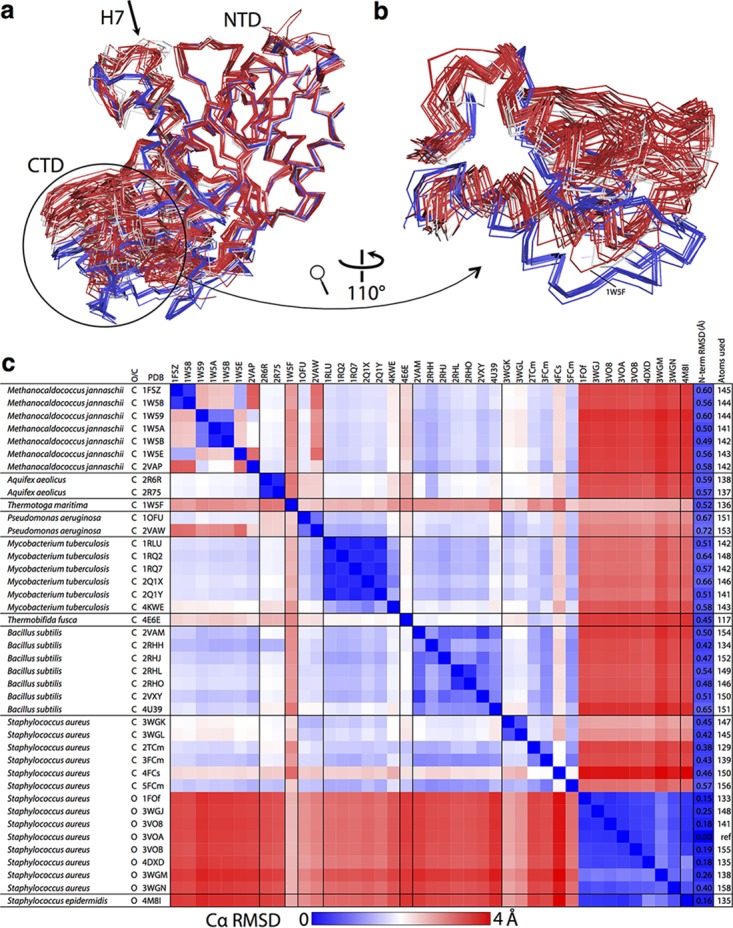
All FtsZ structures can be grouped into two conformations, open and closed. (A) All previous FtsZ structures were obtained from the PDB as listed in panel C. Chain A was extracted from each downloaded structure and the five structures determined here and aligned with the NTD (residues 12 to 176) of 3VOA by using the PyMOL align command, which matches residues via sequence and then minimizes the RMSD with five cycles of outlier rejection, except for PDB ID 1W5F and our structure 4FCs, which are both domain swapped. In these cases, a pseudomonomer was generated for each. Also, *S. aureus* apo structures (PDB IDs 3VO9 and 3VPA), which have a very different conformation (21), were excluded. N- and C-terminal extensions were removed, and the aligned structures are shown in ribbon representations from the same view as in Fig. 2B. Closed structures are red, except for closed *S. aureus* structures, which are in white, and open structures are blue. The structural conservation of FtsZ proteins is clear from the quality of alignment at the NTD (the outlier-excluded RMSDs, and the number of Cα used is shown in the last two columns of panel C). The two groups of structures can be distinguished because of the relative motion of the CTD—the open blue structures are separated from the closed white and red ones. (B) The discrete distinction between the two groups is made clearer by zooming into the CTD as indicated. (C) Cα RMSDs were calculated for all structures versus all structures, by using the PyMOL align command with zero cycles of outlier rejection (i.e., all residues matched via sequence are included in the RMSD calculation). The RMSD for each pair of structures is indicated with a linear three-color gradient as indicated below the matrix. Within each species, sets of highly similar structures are found (blue squares on the diagonal filling the black lines), with the exception of *S. aureus*, where the two conformations, open and closed, align poorly. The *S. aureus* closed structures are more similar to FtsZ proteins from other species than they are to open *S. aureus* structures, indicating that all existing non-*S*. *aureus* FtsZ structures are in similar, closed, conformations.

On the basis of previous work that established that all FtsZ structures are broadly similar (33), we decided to compare all FtsZ structures to all others, including the ones presented here. The striking similarity of all FtsZ structures, except SaFtsZ open forms, is illustrated in Fig. 3. There are many interesting results in Fig. 3C, but the most significant is the fact that SaFtsZ closed forms have a conformation more similar to that of other FtsZ proteins, even evolutionarily distant archaeal ones, than to that of SaFtsZ open structures. The most obvious outlier to the overall trend is PDB ID 1W5F, the previously published structure of a domain-swapped FtsZ from the extremophile bacterium *Thermotoga maritima*. In the structural alignment in Fig. 3A and B, the 1W5F structure can be easily identified, as it falls approximately between the two clusters. Also, our domain-swapped 4FCs structure aligns relatively poorly with the other closed structures, although it is much more similar to closed structures than to open ones. Both cases are perhaps unsurprising, as domain swapping will clearly impose additional constraints on the conformational freedom of the protein. In the case of 1W5F, the two swapped monomers contact one another via their CTDs, so it is unlikely to represent a functionally relevant intermediate form.

We conclude that SaFtsZ exists in two distinct conformations, open and closed, and the closed form is much more similar to all other FtsZ structures than to the SaFtsZ open conformation.

### Crystal structures of polymerization-compromised SaFtsZ mutants reveal structural features of the conformational switch.

**FIG 4:**
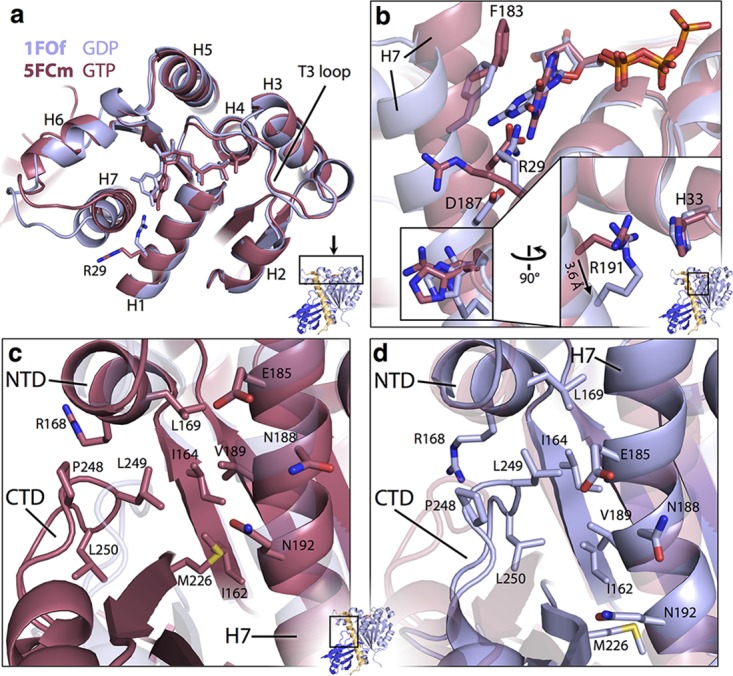
Atomic details of the SaFtsZ regions allowing the conformational switch. Cartoon representation of structures 1FOf (open, blue) and 5FCm (closed, red) are shown superposed after alignment on the NTD. Nucleotides and labeled residues are shown as sticks. Noncarbon atoms are colored by element, except in panel A. The viewpoint is indicated in small cartoons with coloring as in Fig. 2. (A) Top view of FtsZ NTD. Helices are numbered. Note the very minimal rearrangements in this region after both a conformational switch and nucleotide hydrolysis. (B) View of the top of H-7 and into the nucleotide binding pocket. Cartoons are semitransparent. The inset is on same scale and shows the molecule rotated 90° as indicated. The shift of R191 Cα is 3.6 Å. Note the rearrangement of individual side chains between conformations. (C, D) Identical views of the three-way interaction among the NTD, the CTD, and H7 at the top of H-7. 1FOf is semitransparent in panel C with no side chains shown, in panel D 5FCm is semitransparent. Identical side chains are shown in both. The three-way interaction is different in each conformation, but solvent is excluded from the hydrophobic core in both cases.

To exist in two stable globular conformations, FtsZ must have structural features that are rearranged during switching. These features are best visualized by using computational morph interpolations (34) between the structures shown in Fig. 2, as in Movie S1. The large rotation of the CTD versus the NTD requires local rearrangement of residues to maintain hydrophobic contacts, the side chain solvation state, and generally favorable intramolecular interactions. The degree of local rearrangement required is reduced by the movement of H-7, which moves as to stagger rearrangement across the interior faces of the two domains. Displacement of the C-terminal portion of H-7 versus the CTD is facilitated by a large hydrophobic region on the interior face of the CTD’s beta sheet being able to rotate against hydrophobic residues on H-7. Side chain rearrangements here are relatively minor. Regions of greater rearrangement around H-7 are highlighted in Fig. 4, where structures 1FOf and 5FCm are compared (and measurements refer to this pair), although all of the changes discussed are similar in any of our pairs of open and closed structures. Figure 4A and B show rearrangements at the NTD-facing side of H-7, around the nucleotide pocket. Notably, when shifting from closed to open, Arg 29 moves from the solvent-exposed side of H-7 to become slotted between H-7 and the NTD in the open state (a 6.5-Å displacement of the guanidinium carbon), interacting directly with both guanosine and Asp 187 (on H-7, in the closed state itself interacting with the base). Fittingly, R29-E187 is a conserved ion pair in many FtsZ proteins (29). Despite the downward movement of H-7, Phe 183 (also on H-7) maintains favorable π stacking with guanosine, because the base rotates around the C1'-N9 bond. The switch from closed to open leads to disruption of ionic interactions across the C-terminal part of H-7 and NTD residues; however, a subtle rearrangement takes place to maintain a base-base interaction (35) between Arg 191 and His 33 (Fig. 4B, inset): the flexibility and length of the Arg side chain are used to allow the head group to remain almost fixed despite the ~4-Å movement of the Cα atom.

10.1128/mBio.00254-17.3 MOVIE S1  The SaFtsZ conformational switch. Shown is a morph interpolation between SaFtsZ structures 1FOf and 3FCm from two angles with and without side chains. Download MOVIE S1, MOV file, 5.8 MB. Copyright © 2017 Wagstaff et al. 2017 Wagstaff et al. This content is distributed under the terms of the Creative Commons Attribution 4.0 International license.

Figure 4C and D illustrate rearrangement in the three-way interactions among the N-terminal part of H-7, the NTD, and the CTD. While the residues from the NTD involved in the three-way contact remain relatively fixed in the shift from closed to open, e.g., Leu 169, residues from the CTD beta sheet, and H-7 move downward in a coordinated fashion and a loop (residues 246 to 258) from the CTD loosens, allowing residues 248 to 250 to move toward H-7, maintaining solvent exclusion from the hydrophobic pocket.

The conformational switch does not appear to involve structural changes around the phosphate-binding region of the nucleotide-binding pocket (Fig. 4A). In particular, the T3 loop can be ordered in all permutations of nucleotide (GDP/GTP or GTPγS) and conformation (open or closed) (1FOf, 3FCm, 5FCm, PDB ID 3WGN) and can even be disordered when GTP bound (2TCm). These observations appear inconsistent with simple mechanisms of hydrolysis-associated FtsZ monomer conformational change (30, 36), although the terminal phosphate may modulate protein dynamics in a nonobvious way.

### Closed forms of FtsZ proteins correspond to the free monomer, and open forms correspond to the polymerized subunit.

**FIG 5:**
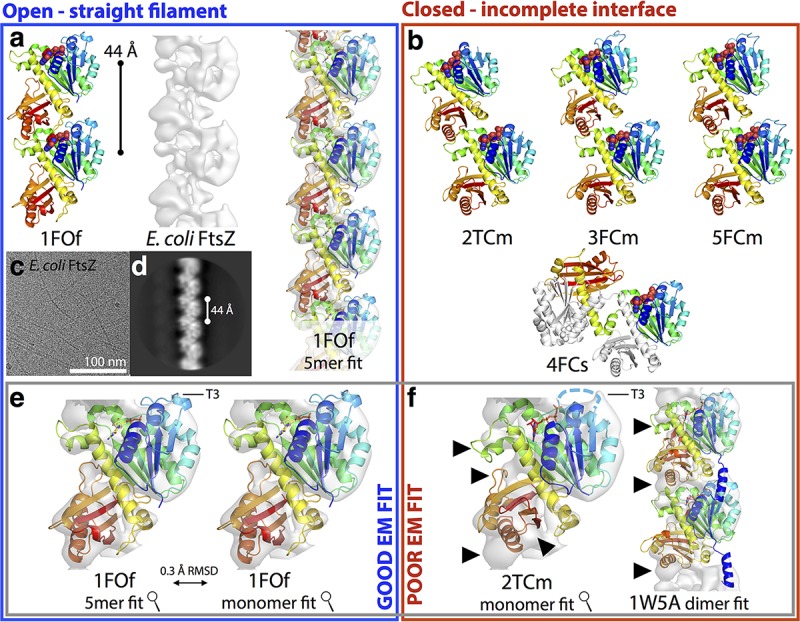
The closed conformation corresponds to the monomeric state of FtsZ, and the open conformation corresponds to the filament, including in *E. coli*. (A, B) FtsZ pairs were extracted from crystal lattices as described in the text. Structures are shown in cartoon representations, and each chain is rainbow colored blue to red, from the N terminus to the C terminus. Nucleotide atoms are colored by element. In each case, the view is from the same orientation after the lower molecule is aligned with the NTD of the lower subunit from 1FOf. 4FCs is shown with one chain colored and the other white to highlight the domain swap. EcFtsZ filament cryoEM density is also shown in panel A at a threshold of 7.5 σ (middle) and also with a 1FOf filament fitted into it (right). Open structures can be arranged to have 44-Å repeats by using favorable tubulin-like interfaces. Closed structures (B) have smaller, incomplete, intersubunit interfaces within crystals and cannot be sensibly arranged to produce straight filaments with a 44-Å repeat. (C) Typical micrograph of frozen-hydrated EcFtsZ GMPCPP filaments. Curved, straight, single, and double/bundled filaments are shown. (D) Representative EcFtsz filament 2D class produced by RELION. (E, F) FtsZ structures as indicated were fitted into the EcFtsZ cryoEM density with the CHIMERA volume viewer fitting tool. The flexible T3 loop region is indicated. (E) A 1FOf 5-mer fits very well into the density, as does a 1FOf monomer, with both fits extremely similar. RMSD is for the middle subunit in rigidly fitted 5-mer and monomer molecules fitted into the middle subunit density. (F) Closed structures do not fit well into the electron density, certainly not so that a repeating filament can be constructed. Some regions of poor fit are indicated by arrowheads. 2TCm was fitted by using only the NTD, which fit into the same position as the open-structure NTDs. EM, electron microscopic.

We have classified our five polymerization-compromised mutant SaFtsZ structures as having a closed or open conformation, but they can also be grouped according to how they are found in relation to other molecules in the crystal. SaFtsZs in 1FOf and in previously published open forms (PDB IDs 3WGM, 3VOA, 3VOB, 3VO8, and 4DXD) are arranged in straight, single, tubulin-like filaments extending through the crystal, indicated in our naming scheme by the second, lowercase, letter f in 1FOf. Adjacent subunits from 1FOf, extracted from the crystal lattice (constructed by using crystallographic symmetry operators) are shown in Fig. 5A. As in tubulin, the nucleotide forms a large part of the interface between subunits, and it is thought that nucleotide hydrolysis is used to modulate interface affinity (37). These crystalline filaments have a 44-Å repeat that corresponds well to repeat intervals seen in negatively stained FtsZ filaments from a number of species. As a result, it has been hypothesized that the 1FOf-like crystal filaments resemble soluble FtsZ filaments.

As discussed, previous work generated SaFtsZ structures in the closed form by extensive alteration of the T7 loop (PDB IDs 3WGL and 3WGK). These structures contain SaFtsZ proteins that are clearly in the closed conformation (Fig. 2D and 3) and are arranged in straight filaments in the crystal. However, these filaments are not the same as the open-form filaments, with a much smaller interface buried surface area (BSA) of ~700 Å^2^, compared to ~1,200 Å^2^ for 1FOf and PDB ID 3VOA (calculated with the PDBe PISA server [38]), and a repeat of 45 Å. A dimer from a 3WGL pseudofilament is shown in Fig. S1. The longitudinal contact is made between residues from the bottom subunit at the N terminus of H5 and the preceding loop (including residue F138), and the loop between H6 and H7. From the top subunit, the T7 loop (replaced in these structures), one face of S9, and the loop at the N terminus of H10 are involved. Interaction does not involve any of the residues on the other side of the interface, toward the phosphates of the nucleotide.

10.1128/mBio.00254-17.1 FIG S1  Crystallized pairs of FtsZ molecules show the difference in longitudinal interactions between open and closed structures. Pairs of FtsZ proteins were extracted from the crystal structures indicated, as for Fig. 5. Structures are shown in cartoon representations where each chain is rainbow colored blue to red from the N terminus to the C terminus. Nucleotide atoms are colored by element. In each case, the view is from the same orientation after the lower molecule is aligned with the NTD of the lower subunit from the 1FOf dimer. Download FIG S1, TIF file, 7.7 MB. Copyright © 2017 Wagstaff et al. 2017 Wagstaff et al. This content is distributed under the terms of the Creative Commons Attribution 4.0 International license.

In contrast, we present four crystal forms where, for the first time, SaFtsZ is not arranged in straight, infinitely long filaments. In three of these, 2TCm, 3FCm, and 5FCm, we find in each case that one of the molecules in the asymmetric unit (ASU) forms what looks superficially like a filament interface via its top face, and the other molecule in the ASU equivalently contributes a bottom face to another pseudointerface. The crystals are therefore composed of pairs of poorly interacting FtsZ proteins (shown in Fig. 5B) that pack via further crystal contacts that do not resemble interfaces in any way. The pseudointerfaces have a subunit-subunit BSA of 670 to 800 Å^2^ and look similar to the interfaces seen in the closed T7 mutant structures, only including residues from one side of the top face.

We know that our mutations compromise filament formation (Fig. 1), and we obtained crystals where these mutant SaFtsZ proteins adopt the closed conformation and fail to form bona fide interfaces. Hence, we propose that these closed forms correspond to the conformation of monomeric SaFtsZ in solution. The pseudointerface seen is perhaps best seen in this context as a consequence of crystallization, which uses conditions that enhance protein-protein interactions, and not a cause. If the protein will crystallize, it is extremely likely that one of the major crystal contacts will imitate the longitudinal interface, as the interface regions are most likely sticky and more complementary than other surface regions. However, we cannot formally rule out the possibility that this minimal interface (*in silico* repetition of which generates a highly curved filament) is a functionally relevant and stable way for FtsZ proteins to interact in solution.

The fact that the phosphate end of the interface is not formed in any of the F138A closed-form crystals supports the idea that it is the closed conformation that is not compatible with the formation of bona fide interfaces, not the mutation *per se*—because the F138A mutation is within the pseudointerface.

The fourth closed-form structure, 4FCs, is arranged very differently within the crystal. There are two molecules of FtsZ present in the ASU, although the NTD and CTD of each polypeptide have become disengaged and reformed in pseudocomplete FtsZ molecules with the corresponding domains of crystallographic symmetry-related molecules, a domain swap. The two pseudo-FtsZ proteins formed by each pair of polypeptides in the ASU both adopt the closed conformation (Cα RMSD for comparable atoms in pseudomonomer versus 2TCm 1.0 Å). The domain-swapped FtsZ proteins do not make any crystal contacts that resemble filament interfaces. That a domain swap can happen and that a domain-swapped FtsZ adopts a closed conformation suggest that the two domains have a significant degree of independence and, more importantly here, that when the conformation of SaFtsZ is not modulated by polymerization or pseudointerfaces, the molecule adopts the closed conformation, as suggested previously by molecular dynamic analysis (25).

We are able to significantly bolster the case that polymerization into a straight filament is the driving force for the conformational switch we observe in crystal structures by presenting a medium (~8-Å)-resolution electron cryomicroscopy (cryoEM) structure of wild-type, FL *E. coli* FtsZ (EcFtsZ) straight filaments in Fig. 5A, C, and D. These data suffer from information anisotropy due to the poor recovery of certain filament orientations in micrographs (Fig. 5C). However, they clearly reveal an FtsZ filament with a 44-Å repeat and a density envelope into which SaFtsZ open-conformation filaments can be fitted very satisfactorily and closed-form structures cannot be fitted well (the *M. jannaschii* closed-conformation dimer structure PDB ID 1W5A is shown fitted, as it has previously been suggested to represent the conformation of FtsZ filaments [32]) (Fig. 5D; see Movie S2). Several secondary structure elements can be unambiguously identified in the reconstruction, including H-7 and, crucially, the planes of both NTD and CTD beta sheets, showing that the molecule is in the open conformation (see Movie S2).

10.1128/mBio.00254-17.4 MOVIE S2  EcFtsZ filaments contain subunits in the open conformation. A SaFtsZ 1FOf filament is shown fitted into the EcFtsZ cryoEM density at 7 sigma contour. 1FOf and 2TCm monomers are shown fitted into the EcFtsZ density at 7 and 9 sigma, with two additional views demonstrating the unambiguous position of both the NTD and CTD beta sheets, showing that the EcFtsZ molecules are in the open conformation. Download MOVIE S2, MOV file, 12.7 MB. Copyright © 2017 Wagstaff et al. 2017 Wagstaff et al. This content is distributed under the terms of the Creative Commons Attribution 4.0 International license.

Given that all FtsZ crystals where bona fide straight filaments are seen in the crystal have FtsZ proteins in the open conformation and that the inverse is true—SaFtsZ filament crystals contain open subunits—and that our intermediate-resolution EcFtsZ cryoEM structure also contains open subunits, we propose that a polymerization-driven conformational switch is a general property of all FtsZ proteins. One of the consequences of such a switch, namely, permitting cooperative assembly of a single-stranded filament, have been discussed previously (24, 27, 29, 39); however, such a switch confers additional surprising properties on a model filament.

### The FtsZ conformational switch between monomer and filament provides filament end asymmetry necessary for treadmilling.

While we were preparing this report, it became clear to us that theoretical considerations of treadmilling can be fraught with intellectual traps. Having run the gauntlet of these potential pitfalls, we present a simplified yet robust schema to explain how a polymerization-associated conformational switch provides the end asymmetry necessary for treadmilling within a single-stranded filament. We focus on the specific case where the nucleotide forms part of the filament interface (i.e., in a tubulin-like fashion). In these, solvent-exposed NDPs (nucleoside diphosphates) are quickly exchanged with NTPs (nucleoside triphosphates), NTP hydrolysis is not immediate, and NTP interfaces are stronger than NDP interfaces; although many of the conclusions are the same for filaments where a nucleotide is buried inside subunits and has an allosteric effect on interface strength (i.e., in an actin-like fashion).

Treadmilling of cytoskeletal filaments is a useful dynamic property. Treadmilling filaments can be used to push or pull molecules in the cell without motor proteins as long as end-tracking mechanisms or cofactors exist, and these behaviors can be made switchable with high flux through the filament (e.g., in eukaryotic anaphase microtubules [40]). Recently, it has been shown that individual FtsZ filaments can treadmill *in vitro* with FtsA (15) and also alone (16) and that FtsA/Z treadmilling in cells guides septal cell wall remodeling (17, 18). However, as has been noted previously (41), a single-stranded filament with the above properties and rigid subunits, without conformational changes, cannot do robust treadmilling.

**FIG 6:**
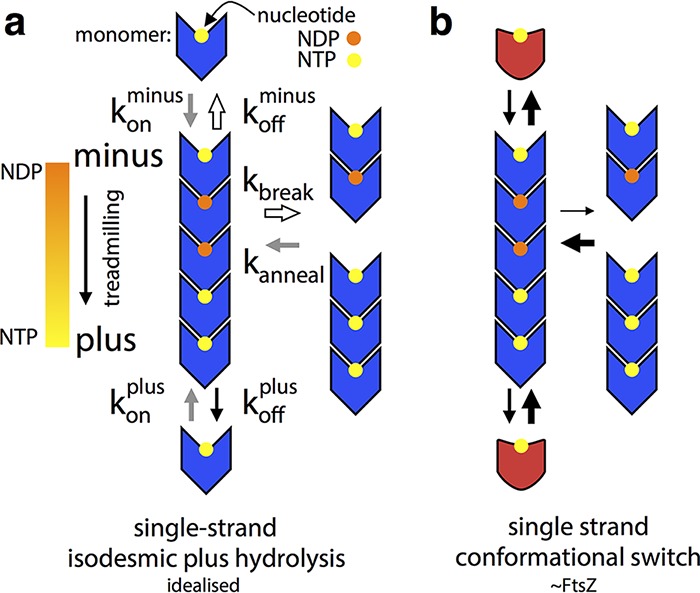
FtsZ’s polymerization-associated conformational switch allows treadmilling of single-stranded filaments. Black arrows indicate rates roughly in proportion to their width, and similarly colored arrows in panel A indicate rates that are exactly equivalent. See the text for a discussion of the limitations and assumptions of these simplified models, particularly regarding implied orientation of molecules. (A) An idealized rigid (lacking a conformational switch), tubulin-like, filament-forming protein, for which addition or loss of a given nucleotide:monomer complex is isodesmic. This filament cannot do robust treadmilling, as breakage is the same as minus end subunit loss, and it cannot couple structural and kinetic polarity. (B) A single-stranded version of panel A with a polymerization-associated conformational switch (between blue and red forms) able to treadmill robustly and with coupled kinetic and structural polarities. The conformational switch allows filament breakage and subunit loss from ends to be different and for the stereochemistry of subunit addition at either end to be different, meaning that addition will take place at different rates in a manner defined by structural polarity.

Such a hypothetical filament with rigid subunits is shown in Fig. 6A. Note that the location (top/bottom) of nucleotide binding to the monomer is not important. This filament treadmills if a nucleotide gradient along the filament exists, and the kinetic plus end (net growth) will be at the end with more NTP. On rates cannot differ at the two ends because they are the same reaction, but the off rate at the minus (NDP) end will be greater than at the plus end, so a situation of net growth at one end and net shrinkage at the other can be produced at certain monomer concentrations (addition reaction is first order with respect to monomer, and loss is zero order). This does not represent robust treadmilling, however, as breakdown of terminal GDP interfaces is equivalent to breakdown of a GDP interface anywhere in the filament, and these processes will occur at the same rate, as they are all zero order. As noted previously (42), filament breakage and annealing could be an important facet of dynamics, but the filament in Fig. 6A has a more fundamental limitation of its biological usefulness in that the direction of treadmilling is determined entirely by the history of the filament (the direction of the initial NTP-NDP gradient), so there is no coupling of kinetic and structural polarity, and the same filament could just as easily treadmill in either structure-defined direction.

Coupling of kinetic and structural polarity requires subunit addition and/or loss to proceed via different stereochemical pathways at each structure-defined end of the filament. This is not the case in Fig. 6A; the difference in off rates (the kinetic polarity) is set by the nucleotide gradient and not by the structural polarity, and we have already seen that there can be no difference in the on rates at either end. Filament systems can generate different stereochemistry for subunit addition at either end by being multistranded and having staggered subunits that undergo a conformational change, such as actin (43), or by using a longitudinal hooking mechanism, such as TubZ (44).

**FIG 7:**
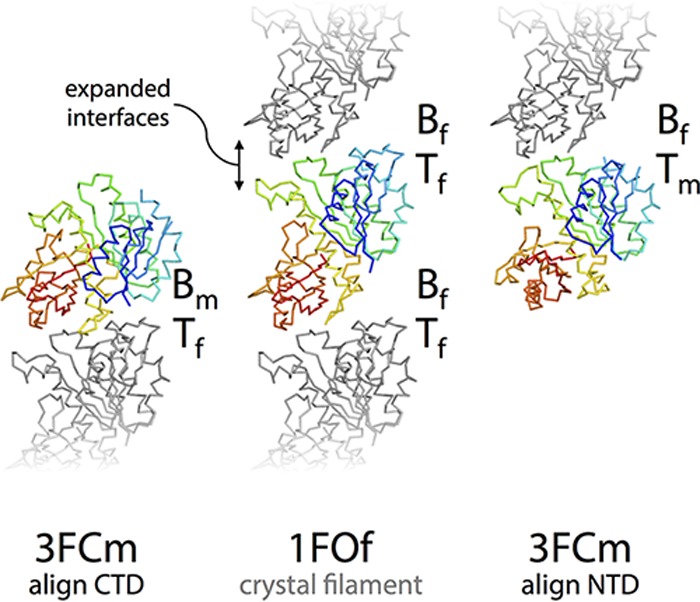
A polymerization-associated conformational switch generates asymmetry between filament end interfaces. (Middle) Three molecules from the open-form 1FOf crystal filament, slightly separated for clarity, are shown as Cα ribbons. The middle subunit is rainbow colored blue to red from the N terminus to the C terminus, and the top and bottom subunits are gray (right, left). The middle subunit is replaced with a closed-form 3FCm molecule aligned with the middle subunit NTD (right) or CTD (left), as indicated by the arrows. The different pairs of approaching surfaces are labeled B/*T*_m/f_ for bottom/top monomer/filament. These modeled closed and open interfaces do not represent the transition state of subunit addition at either end of a filament (or even any position on the reaction pathway), but they illustrate the fact that the conformational switch will necessarily lead to stereochemically different reaction pathways at each end that allow the two ends to have different rates of subunit addition, linking structural and kinetic polarity.

Figure 6B shows our model of how a single filament very similar to the case in Fig. 6A can couple its structural polarity to a defined kinetic polarity and thus usefully, and robustly, treadmill. The crucial difference between Fig. 6A and B is the existence of a polymerization-associated conformational switch; i.e., subunits are no longer rigid but can exist in one of two conformations, one form associated with the polymer and the other adopted in the free monomer. The free-energy cost of the conformational switch from closed to open is paid by binding to a filament end and in the other direction through nucleotide hydrolysis and exchange that makes the longitudinal NDP intersubunit interface unfavorable. Although the formation of an NTP interface at either structurally defined end has the same net energy change, the reaction pathways are stereochemically different and will occur at different rates because two different pairs of molecular surfaces are involved in each case initially. This difference is illustrated in the context of our structures in Fig. 7, but note that we are not making a prediction about which end of a single-stranded FtsZ filament is the kinetic plus end.

Importantly, the scheme in Fig. 6B also allows breakage at NDP interfaces within the filament to be different from loss of NDP subunits from each end; NDP interfaces in the filament are stronger because the energetic cost of losing subunits from ends only is paid for by the favorable switch to the monomer conformation, which the “new” end subunits of two halves of a broken filament cannot do because they remain in filaments through one remaining interface. It is especially important to note here that the scheme in Fig. 6B can also be drawn with a nucleotide on the other side of the monomer; i.e., again we are not making a prediction about which end of an FtsZ filament is the kinetic plus end—an issue that for FtsZ will need further investigation (42, 45, 46).

### Conclusion.

Here we have shown that FtsZ proteins adopt two different conformations, open and closed. The open form is adopted by FtsZ in straight filaments, and the closed form is adopted by FtsZ monomers. The implication is that the polymerization-associated switch from closed to open is made favorable by the free-energy gain of interface formation of the subunits in the filament. Such a polymerization-associated conformational switch explains how a single-stranded filament can show cooperativity in polymerization and how it can avoid breaking apart when treadmilling. This switch also explains how a single-stranded filament with tubulin-like properties can couple structural and kinetic polarities to enable robust treadmilling, with plus and minus ends being defined by the polarity of the filament.

At this point, it should be highlighted that although single-stranded FtsZ is frequently considered the functional unit of the protein *in vivo*, the potential of the conformational switch to generate end asymmetry could also be exploited in multistranded treadmilling and treadmilling in conjunction with the many FtsZ-interacting proteins *in vivo*. In addition to this, we have not directly addressed the structural basis of filament bending, an outstanding question in the field. Indirectly, cryoEM of EcFtsZ filaments assembled with GMPCPP and frozen after a 20- to 30-s incubation shows some degree of bending in almost all single filaments, and segments from bent filaments are included in the reconstruction showing subunits in the open form, apparently undermining previous ideas that all bent FtsZ filaments are GDP bound and/or in a closed or related conformation.

## MATERIALS AND METHODS

### Cloning, protein expression, and purification of FtsZ.

FL SaFtsZ (UniProt ID FTSZ_STAAU) was amplified by PCR from genomic DNA and cloned into the NdeI and SapI sites of pTXB1 (NEB IMPACT system; NEB, Ipswich, MA), thus generating a C-terminal fusion to the *Mxe* intein/chitin binding domain which self-cleaves upon the addition of dithiothreitol (DTT). PCR mutagenesis with this vector as a template generated T66W and F138A mutant proteins. FL fusion proteins were expressed in *E. coli* BL21(DE3) cells that were grown in LB medium with ampicillin (100 mg/liter) at 37°C with shaking at 200 rpm to an optical density at 600 nm (OD_600_) of 0.6. Cultures were then shifted to 16°C, and after 1 h, expression was induced by the addition of 0.4 mM isopropyl-β-d-thiogalactopyranoside (IPTG) before overnight incubation. Cells were collected by centrifugation and resuspended in buffer FL.A (50 mM HEPES-KOH, 50 mM NaCl, 20 mM EDTA, pH 8.5) with 100 μg/ml lysozyme, 10 μg/ml DNase, and 4 mg/ml phenylmethylsulfonyl fluoride before lysis via two or three passes through a French press. The lysate was clarified by centrifugation at 100,000 × *g* and 4°C for 1 h. Soluble protein was captured on a chitin column (NEB) equilibrated and washed with buffer FL.A. Intein activity and release of the untagged, FL protein was initiated by overnight incubation in buffer FL.B (buffer FL.A with 50 mM DTT) at 4°C, followed by elution. Eluate was further purified by anion-exchange chromatography on a 5-ml HiTrap Q column (GE Healthcare, Little Chalfont, United Kingdom). The column was equilibrated and washed with buffer FL.Q.A (50 mM Tris-HCl, 1 mM EDTA, pH 7.5), and bound protein was eluted with a linear gradient of buffer FL.Q.B (FL.Q.A with 1 M NaCl). Peak fractions were further purified by gel filtration on a 70-ml Sephadex 75 (GE Healthcare) column in buffer FL.GF (20 mM Tris-HCl, 150 mM NaCl, 10% glycerol, pH 8.0). Peak fractions were pooled and concentrated with centrifugal concentrators (Vivaspin; Sartorius, Epsom, United Kingdom) to 5 to 8 mg/ml before being frozen in liquid nitrogen and stored at −80°C. Protein integrity was confirmed by electrospray mass spectrometry.

TR (residues 12 to 316) SaFtsZ (UniProt ID FTSZ_STAAU) was cloned into a pHis17 plasmid derivative with no tag via Gibson assembly techniques. PCR mutagenesis with this vector as a template generated T66W and F138A mutant proteins. TR SaFtsZ proteins were expressed in *E. coli* C41(DE3) cells (Lucigen) grown in 2×TY medium with ampicillin (100 mg/liter) at 37°C with shaking at 200 rpm to an OD_600_ of 0.6. Cultures were then shifted to 16°C, and after 1 h, expression was induced by the addition of 0.5 mM IPTG before overnight incubation. Cells were collected by centrifugation and resuspended in buffer TR.A (50 mM Tris-HCl, 30 mM NaCl, pH 8.0) before lysis by passage through a cell disruptor (Constant Systems, Daventry, United Kingdom) at 25,000 lb/in^2^. The lysate was clarified by centrifugation at 100,000 × *g* and 4°C for 30 min. The soluble fraction was loaded onto a HiTrap Q anion-exchange column (GE Healthcare), washed with buffer TR.Q.A, and eluted with a linear gradient of buffer TR.B (TR.A with 1 M NaCl). Peak fractions were pooled, and protein was precipitated by adding saturated ammonium sulfate to 35%, vol/vol. Precipitated protein was centrifuged at 28,000 × *g* and 4°C for 30 min, and the pellet was resuspended in buffer TR.A. The resuspended protein was further purified by size exclusion chromatography on a HiLoad Sephacryl S300 16/60 column (GE Healthcare) in buffer TR.A. Peak fractions were pooled and concentrated to 15 to 25 mg/ml with centrifugal concentrators (Vivaspin; Sartorius) before being frozen in liquid nitrogen and stored at −80°C. Protein integrity was confirmed by electrospray mass spectrometry.

FL, untagged EcFtsZ (UniProt ID FTSZ_ECOLI) was cloned into the BamH/NdeI sites of pET9a (Novagen) with no tag. Purification of EcFtsZ was done by a modified version of established protocols (47). Protein was expressed in *E. coli* C41(DE3) cells (Lucigen) grown in 2×TY medium with kanamycin (50 mg/liter) at 37°C with shaking at 200 rpm to an OD_600_ of 0.6. Cultures were then shifted to 20°C, and after 1 h, expression was induced by the addition of 0.5 mM IPTG before overnight incubation. Cells were collected by centrifugation and resuspended in buffer PEM (50 mM PIPES-KOH, 5 mM MgCl_2_, 1 mM EDTA, pH 6.5) before lysis by passage through a cell disruptor (Constant Systems) at 25,000 lb/in^2^. The lysate was clarified by centrifugation at 100,000 × *g* and 4°C for 30 min. FtsZ was separated by GTP- and calcium-induced precipitation. Lysate was adjusted to pH 7 with acetic acid, and then GTP and CaCl_2_ were added to 1 and 20 mM, respectively. This mixture was then centrifuged at 11,000 × *g* and 4°C for 15 min. The pellet, containing FtsZ, was resuspended in buffer PEM, and insoluble debris was removed by centrifugation at 100,000 × *g* and 4°C for 30 min. FtsZ was further purified by anion-exchange chromatography over a Mono Q 4.6/100 (1.7 ml) PE column (GE Healthcare) equilibrated and washed with buffer ECZ.Q.A (50 mM Tris-HCl, 50 mM KCl, 1 mM EDTA, 10% glycerol, pH 8.0), and bound protein was eluted with a linear gradient of buffer ECZ.Q.B (buffer ECZ.Q.A with 1 M KCl). Peak fractions were pooled and concentrated to 20 mg/ml with centrifugal concentrators (Vivaspin; Sartorius) before being frozen in liquid nitrogen and stored at −80°C. Protein integrity was confirmed by electrospray mass spectrometry.

### Sedimentation analysis of assembly.

Samples were prepared in a Thermostat Plus (Eppendorf, Hamburg, Germany) at 25°C, where the protein (10 μM) was equilibrated in buffer HKM (50 mM HEPES-KOH, 100 mM potassium acetate, 5 mM magnesium acetate, pH 7.7). After the addition of 1 mM GTP or 0.1 mM GMPCPP with or without 20 μM PC190723 and 2% DMSO to prevent ligand precipitation, samples were incubated for 20 min at 25°C and then centrifuged at 400,000 × *g* for 20 min at the same temperature in a Beckman TLA 100 rotor. The supernatants were carefully withdrawn, and the pellets were resuspended in the same volume of buffer. Subsequently, pellets and their corresponding supernatants were loaded and run in the same well of an SDS-PAGE gel with a 25-min delay to analyze the amount of protein polymerized in the pellet versus the unassembled fraction in the supernatant.

### GTPase activity assay.

GTP (Sigma, St. Louis, MI), and GMPCPP (Jena Bioscience, Jena, Germany) hydrolysis by FL SaFtsZ proteins was monitored by detecting the release of inorganic phosphate with the malachite green assay (48) in samples containing 10 or 20 μM protein in buffer HKM at 25°C. When included, PC190723 was at a concentration of 20 μM, and samples contained 2% DMSO to prevent ligand precipitation.

### Negative-stain electron microscopy.

FL wild-type and mutant SaFtsZ proteins were visualized by negative-stain electron microscopy. About 20 μl of sample prepared as for sedimentation analysis of assembly (except that the protein concentration was 20 μM) was applied to a Formvar- and carbon-coated copper grid, incubated for 1 min, and then stained with 2% (wt/vol) uranyl acetate in water. Images were taken at several magnifications with a JEOL 1200 EX-II microscope operated at 100 kV and equipped with a Gatan charge-coupled device camera.

### Crystallization.

Crystallization conditions were found with our in-house high-throughput crystallization platform by mixing 100 nl of TR SaFtsZ T66W or F138A solution at 5 or 10 mg/ml with GTP at 10 mM with 100 nl of 1,920 different crystallization reagents in Medical Research Council vapor diffusion sitting-drop crystallization plates. Conditions yielding crystals were optimized, and crystals from either the initial screenings or subsequent optimization were selected for data collection. For the conditions that give the crystals for which structures are presented, see Table S1.

### Crystallographic data collection and structure determination.

Diffraction images were collected from single frozen crystals at 100 K at beamlines at either the Diamond Light Source (Harwell, United Kingdom) or the European Synchrotron Radiation Facility (Grenoble, France) as indicated in Table S1. Diffraction images were processed with XDS, POINTLESS, and scala software. Initial phases were determined by molecular replacement with PHASER by using the search models indicated in Table S1. Models were rebuilt manually with MAIN and refined with refmac and PHENIX.REFINE. Ramachandran plots and MOLPROBITY statistics were used to validate the structures.

### Structure of EcFtsZ filament determined by cryoEM.

For cryoEM, EcFtsZ was prepared at 0.5 mg/ml of a mixture of 50 mM HEPES-KOH, 100 mM potassium acetate, and 5 mM magnesium acetate, pH 7.7, at 20°C. GMPCPP was added to 0.1 mM. A 2.5-μl sample was applied to a freshly glow-discharged Quantifoil Cu R2/2 200-mesh grid and plunge frozen with a Vitrobot Mark III (FEI Company, OR) in liquid ethane maintained at 93.0 K with an ethane cryostat (49). The Vitrobot chamber temperature and humidity were set to 10°C and 100%, respectively. Micrograph movies of FtsZ filaments were collected with an FEI Tecnai G2 Polara microscope operating at 300 kV. Data were acquired on a Falcon III direct electron detector prototype at a calibrated pixel size of 1.34 Å and an approximate total dose of 40 e^−^/Å^2^ with the automated acquisition software EPU (FEI Company). A total of 3,688 movies were collected at −1- to −4-μm defocus in 46 frames during each 1.5-s exposure. All image processing was carried out within RELION 2.0 (50). Micrograph movies were motion corrected with MotionCor2 (51) with five-by-five patches and a grouping of 10 frames. Helical autopicking in RELION was used to find segments along the filaments at 4.3-nm intervals with confidence. Boxes of 190 by 190 pixels were extracted around each segment. After two-dimensional (2D) classification, three-dimensional (3D) autorefinement of the remaining 943,277 filament segments was performed in RELION by using helical reconstruction options against an atomic protofilament model derived from PDB ID 3VO8 filtered to 20 Å. The resulting two half maps were used in postprocessing to sharpen the map (B factor, −360 Å^−1^) and to obtain a gold standard Fourier shell correlation (FSC)-based resolution estimate of 6.7 Å (0.143 FSC criterion). In the absence of an EcFtsZ crystal structure, CHIMERA was used to determine that the SaFtsZ filament structure, showing the open conformation, fits very well into the *E. coli* filament density, as opposed to any other structure containing closed conformations.

### Structural figures.

All structural figures were prepared in PyMOL (Schrödinger, Inc.), with some density volume operations carried out in CHIMERA (52).

### Availability of data.

The crystal structures determined in this study are available in the Protein Data Bank under IDs 5MN4, 5MN5, 5MN6, 5MN7, and 5MN8.
