# Supplementary material for: A Polymerization-Associated Structural Switch in FtsZ That Enables Treadmilling of Model Filaments
Source: mBio. 2017 May 2;8(3):e00254-17. doi: 10.1128/mBio.00254-17 (PMC5414002; doi:10.1128/mBio.00254-17)
Supplement: TABLE S1 [file mbo002173299st1.docx]

Table S1. Crystallographic and cryoEM data

| **Statistics** | **1FOf**  FtsZm3-SaF138A17b12_9 | **2TCm**  3D9_T66W_E8 | **3FCm**  FtsZm3-SaF138A17b4_5 | **4FCs**  11_1_LMB19_H4_d1_3_1 | **5FCm**  11_1_LMB9_C8_d2_2_1 | **cryoEM** |
| --- | --- | --- | --- | --- | --- | --- |
| **Sample** | *Staphylococcus aureus* FtsZ (F138A) | *Staphylococcus aureus* FtsZ (T66W) | *Staphylococcus aureus* FtsZ (F138A) | *Staphylococcus aureus* FtsZ (F138A) | *Staphylococcus aureus* FtsZ (F138A) | *Escherichia coli* FtsZ |
| UniProt ID | FTSZ_STAAU | FTSZ_STAAU | FTSZ_STAAU | FTSZ_STAAU | FTSZ_STAAU | FTSZ_ECOLI |
| Constructs  Crystallisation/grids | 12-316, no tag  F138A mutation  10 mg/ml; 12.5 % w/v PEG 1k, 12.5 % w/v PEG 3350, 12.5 % v/v MPD, 0.1 M bicine/Tris pH 8.5, 0.03 M NaF, NaBr and NaI; cryo 30 % ethylene glycol | 12-316, no tag  T66W mutation  10 mg/ml; LiCl 1.136 M, PEG 6,000 31.4 %, 0.1 M MES pH 6; cryo 20 % PEG 200 | 12-316, no tag  F138A mutation  10 mg/ml; 12.5 % w/v PEG 3,350, 12.5 % w/v PEG 1k, 12.5 % v/v MPD, pH 6.5, 0.1M MES/imidazole, 0.03 M NaF, NaBr and NaI; cryo 30 % ethylene glycol | 12-316, no tag  F138A mutation  10 mg/ml; 40% PEG monomethyl ether 350, 0.05 M MES pH 6; cryo 20 % glycerol | 12-316, no tag  F138A mutation  5 mg/ml; 1.6 M ammmonium sulfate, 0.5 M LiCl; cryo 20 % glycerol | full length, no tag  0.5 mg/ml in 50 mM HEPES/KOH, 100 mM K-acetate, 5 mM Mg-acetate, pH 7.7; Quantifoil Cu R2/2 200 mesh |
| **Method**  **Data collection** | crystallography  molecular replacement  model 3VO8 | crystallography  molecular replacement  model 3WGL | crystallography  molecular replacement  model 2TCm | crystallography  molecular replacement  model 2TCm | crystallography  molecular replacement  model 2TCm | cryoEM  single particle model 1F0f @ 20 Å |
| Beamline/ microscope | ESRF id23eh1 | ESRF id29 | ESRF id23eh1 | Diamond I04-1 | Diamond I04-1 | FEI Polara G2 |
| Wavelength / energy | 0.93 Å | 0.9792 Å | 0.93 Å | 0.9282 Å | 0.9282 Å | 300 kV |
| **Crystal / helical** |  |  |  |  |  |  |
| Space / point group | C2 | P2_1_2_1_2_1_ | P2_1_2_1_2_1_ | I222 | P3_1_21 |  |
| Cell (Å) | 71.8, 51.1, 88.1, 111° | 43.8, 59.9, 187.7 | 41.1, 68.2, 207.9 | 116.1, 130.0, 134.1 | 69.8, 69.8, 295.5 |  |
| Twist / rise |  |  |  |  |  | 0.0° / 43.8 Å |
| **Data** |  |  |  |  |  |  |
| Resolution (Å) | 1.5 | 2.8 | 3.2 | 3.3 | 3.5 | 7.8 Å, anisotropic |
| Completeness (%)^a^ | 98.5 (97.9) | 98.2 (95.5) | 99.9 (100) | 91.5 (94.3) | 98.3 (99.6) |  |
| Multiplicity^a^ | 3.4 (3.4) | 4.4 (4.3) | 9.9 (8.8) | 3.6 (3.6) | 4.8 (5.1) |  |
| (I) / σ(I) ^a^ | 14.1 (1.7) | 11.4 (3.0) | 10.6 (1.7) | 11.6 (1.9) | 6.8 (1.4) |  |
| R_merge_^a^ | 0.045 (0.652) | 0.109 (0.554) | 0.132 (1.107) | 0.098 (0.719) | 0.146 (1.226) |  |
| R_pim_^a^  CC1/2  Images, pixel size  Defocus range, dose  Segments | 0.045 (0.411)  0.999 (0.740) | 0.056 (0.286)  0.995 (0.781) | 0.043 (0.389)  0.998 (0.737) | 0.055 (0.408)  0.997 (0.696) | 0.073 (0.606)  0.998 (0.853) | 1834, 1.34 Å  -1.5 - -4.0 µm, 40 e/Å^2^  511,000 |
| **Refinement** |  |  |  |  |  |  |
| R / R_free_^b^ | 0.178 / 0.212 | 0.217 / 0.263 | 0.216 / 0.299 | 0.211 / 0.268 | 0.2649 / 0.3137 | not refined |
| FSC (REFMAC)  Models  Bond length rmsd (Å) | 1 chain/ASU: 12-315, 1 GDP, 1 MPD, 251 waters  0.009 | 2 chains/ASU: A:13-62, 74-315, B:13-62, 74-202, 209-315, 2 GTP, no waters  0.006 | 2 chains/ASU: A:13-201, 209-315, B:12-315 2 GDP, no waters  0.010 | 2 chains/ASU: A/B:13-315,  2 GTP, 2 Mg, no waters  0.002 | 2 chains/ASU: A/B:13-202,207-315,  2 GTP, no waters  0.002 |  |
| Bond angle rmsd (º) | 1.158 | 0.946 | 1.573 | 0.505 | 0.575 |  |
| Favoured (%)^c^ | 99.6 | 99.6 | 100 | 99.8 | 99.8 |  |
| Disallowed (%)^c^  MOLPROBITY score | 0.4  93th percentile | 0 99th percentile | 0  98th percentile | 0.2  100th percentile | 0.2  100th percentile |  |
| **PDB ID** | **5MN4** | **5MN5** | **5MN6** | **5MN7** | **5MN8** |  |

^a^ Values in parentheses refer to the highest recorded resolution shell. ^b^ 5% of reflections were randomly selected before refinement. c Percentage of residues in the Ramachandran plot (PROCHECK 'most favoured' and 'additionally allowed' added together).
