# Supplementary figures and images for: A Polymerization-Associated Structural Switch in FtsZ That Enables Treadmilling of Model Filaments
Source: mBio. 2017 May 2;8(3):e00254-17. doi: 10.1128/mBio.00254-17 (PMC5414002; doi:10.1128/mBio.00254-17)

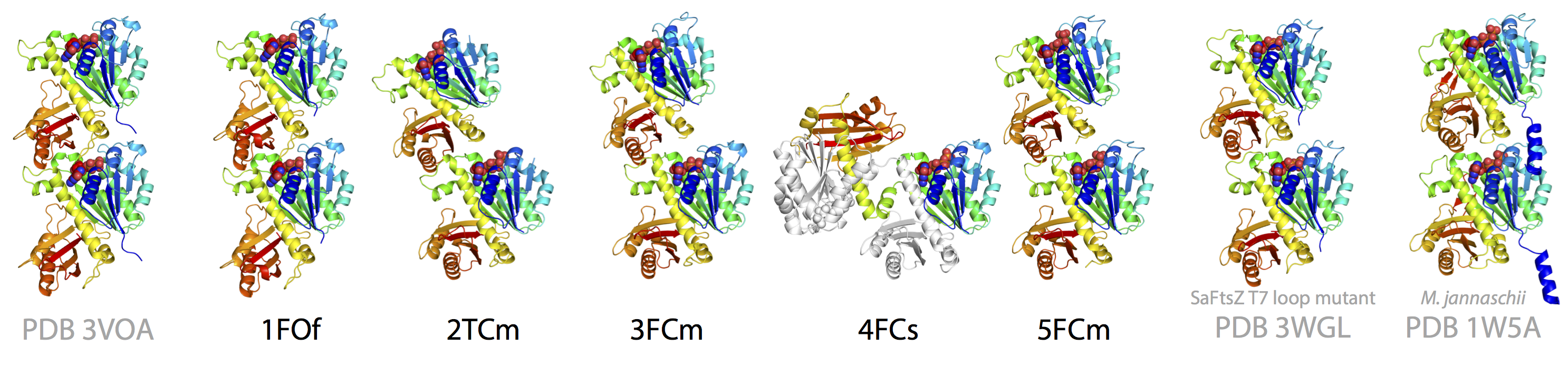

Supplement: FIG S1 [file mbo002173299sf1.tif]
